# Effects of a Patient Activation Tool on Decision Making Between Surgery and Nonoperative Management for Pediatric Appendicitis: A Randomized Clinical Trial

**DOI:** 10.1001/jamanetworkopen.2019.5009

**Published:** 2019-06-07

**Authors:** Peter C. Minneci, Jennifer N. Cooper, Karen Leonhart, Kristine Nacion, Jason Sulkowski, Kyle Porter, Lai Wei, Katherine J. Deans

**Affiliations:** 1 Center for Surgical Outcomes Research, The Research Institute at Nationwide Children’s Hospital, Columbus, Ohio; 2 Center for Biostatistics, Department of Biomedical Informatics, The Ohio State University College of Medicine, Columbus

## Abstract

**Importance:**

Strategies to activate and engage patients and caregivers in shared decision making in the acute care setting may result in improved outcomes.

**Objective:**

To determine whether a patient activation tool (PAT) can improve decision-making and patient-centered outcomes among pediatric patients and their caregivers who choose between surgery and nonoperative management for their child’s appendicitis.

**Design, Setting, and Participants:**

This single-blind, randomized clinical trial collected data from a single tertiary children’s hospital from March 1, 2014, through April 30, 2016, with 1-year follow-up completed on May 1, 2017. Two hundred of 236 eligible children and adolescents aged 7 to 17 years with uncomplicated appendicitis enrolled with their caregivers. After receiving the randomized clinical intervention, caregivers chose surgery or nonoperative management. Data were analyzed from March 1, 2014, through May 1, 2017.

**Interventions:**

Randomization to a scripted standardized surgical consultation that emphasized patient choice or a scripted standardized surgical consultation plus the PAT (a tablet-based tool that presents each treatment, encourages participation in medical decision making, and aims at alleviating decisional uncertainty).

**Main Outcomes and Measures:**

Decisional self-efficacy immediately after treatment decision, health care satisfaction at discharge, and disability days for the child at 1-year follow-up.

**Results:**

Among 200 participants (median age, 12 years [interquartile range (IQR), 9-15 years]; 120 [60.0%] male), 98 were randomized to the PAT and 102 to the standardized consultation groups. The percentages choosing nonoperative management were similar (standardized consultation group, 42 of 102 [41.2%]; PAT group, 31 of 98 [31.6%]; *P* = .19). Immediate decisional self-efficacy was similar in the standardized consultation and PAT groups (median score, 100 [IQR, 97.7-100] vs 100 [IQR, 95.5-100]; *P* = .03), which was not significant at the planned significance level of *P* = .02. Total scores on health care satisfaction at discharge were similar (median, 99 [IQR, 94.7-100] vs 98 [IQR, 91.7-100]; *P* = .27). Disability days at 1-year follow-up were also similar (median, 6 [IQR, 2-11] vs 5 [IQR, 2-15]; *P* = .67). No difference in the failure rate of nonoperative management at 1 year (13 of 38 [34.2%] vs 11 of 30 [36.7%]; *P* > .99) or in the rate of complicated appendicitis 30 days after discharge (7 of 68 [10.3%] vs 9 of 71 [12.7%]; *P* = .79) occurred.

**Conclusions and Relevance:**

In this study, a technology-based PAT did not improve measures of decision making for pediatric patients and caregivers needing to make an urgent treatment decision between surgery and nonoperative management for appendicitis. However, the overall high scores in both groups suggest that pediatric patients and caregivers can process information in the acute care setting and effectively participate in an informed shared decision-making process around the need for surgery.

**Trial Registration:**

ClinicalTrials.gov identifier: NCT02110485

## Key Points

**Question:**

Can a patient activation tool improve decision-making and patient-centered outcomes in pediatric patients and their caregivers choosing between surgery and nonoperative management for pediatric appendicitis?

**Findings:**

In this randomized clinical trial that included 200 patient-caregiver dyads, groups randomized to the standardized surgical consultation and the patient activation tool had similarly high scores on immediate decisional self-efficacy and health care satisfaction measures at discharge and no differences in disability days or the failure rate of nonoperative management at 1 year.

**Meaning:**

Although a patient activation tool did not improve measures of decision making, pediatric patients and families can effectively participate in an informed shared decision-making process in the acute care setting.

## Teaser

This randomized clinical trial evaluates the effects of a patient activation tool vs standard consultation on decision making and patient-centered outcomes among pediatric patients with appendicitis and their caregivers who choose surgery or nonoperative management.

## Introduction

Shared decision making with active engagement of patients in health care decisions has been referred to as the pinnacle of patient-centered care.^1^ Essential components of patient activation and engagement include establishing a willingness to take an active role in their care, ensuring confidence in their ability to manage their care, and providing the knowledge and skills necessary to make decisions and manage their care.^2^ Patient activation has been shown to improve healthy behaviors, use of health care services, and the management of chronic illnesses in adults.^3,4,5,6,7,8^ However, the effects of patient activation in the acute care setting have not been well established.

High-acuity illness has been directly associated with patient and caregiver stress, decreased comprehension, and impaired recall.^9,10,11,12,13,14,15^ Emergency surgical interventions present stressful and difficult decisions for patients and caregivers with limited time for patient activation, engagement, and shared decision making. This situation is especially true in vulnerable populations such as children, for whom caregivers are forced to make quick decisions.

This study investigated the effects of patient and caregiver activation on decision-making and patient-centered outcomes for acute appendicitis, a common and urgent surgical disease in children. The choice between appendectomy and nonoperative management with antibiotics alone must balance the unique risk-benefit profile of each treatment with the preferences and values of each individual patient-caregiver dyad. We hypothesized that strategies to activate caregivers in the acute-care setting may result in improved decision-making and patient-centered outcomes without compromising medical outcomes.

## Methods

### Study Design

We performed a randomized clinical trial to compare a patient activation tool (PAT) with standardized surgical consultation in patient-caregiver dyads choosing between nonoperative management and appendectomy for uncomplicated pediatric appendicitis. Based on the findings of previous studies,^16,17^ patients treated at our hospital are offered the choice between surgery and nonoperative management as part of standard clinical practice. A multidisciplinary team of patient, family, and other health care stakeholders was an integral part of the research team throughout this project. This stakeholder team helped us formulate the research question, design the interventions, and perform the study. The stakeholder team composition, engagement, and contributions are detailed in eTable 1 in Supplement 1. This study was approved by the institutional review board of Nationwide Children’s Hospital and follows the Consolidated Standards of Reporting Trials (CONSORT) reporting guideline for randomized clinical trials. Caregivers provided informed written consent, and patients provided assent to participate. Additional details are given in the trial protocol (available in Supplement 2).

### Study Cohort

All children and adolescents aged 7 to 17 years who were treated for appendicitis from March 1, 2014, through April 30, 2016, were screened for eligibility. Inclusion criteria consisted of early appendicitis imaging confirmed by ultrasonography or computed tomography with an appendiceal diameter of less than 1.1 cm and no abscess, fecalith, or phlegmon; white blood cell count of less than 18/μL; C-reactive protein level of less than 4 mg/L (if obtained) (to convert to nanomoles per liter, multiply by 9.524); and focal abdominal pain for no longer than 48 hours. Exclusion criteria consisted of diffuse peritonitis, a positive pregnancy test result, or significant medical or behavioral comorbidities. Eligible patients and their caregivers were invited to participate during surgical evaluation in the emergency department.

Patient-caregiver dyads were randomized to a scripted standardized surgical consultation (control arm) or to a scripted surgical consultation plus the PAT (intervention arm) (eTable 2 and eTable 3 in Supplement 1). Randomization was performed using a web-based process with a varying block size scheme. Both groups were given access to the same information from a research team physician before meeting the attending surgeon. After all questions were answered, the patient-caregiver dyad chose appendectomy or nonoperative management. The clinical team was informed of the treatment decision, and the patient was admitted. Any patient or caregiver who wished to speak with a member of the surgical team before making a decision was provided the opportunity to do so.

### Interventions

#### Standardized Surgical Consultation Group

Patient-caregiver dyads received only scripted verbal information regarding the 2 treatment options using a standardized script. This script was developed with our stakeholders to represent the best available standard of care consultation rather than usual care (eTable 2 and eTable 3 in Supplement 1).

#### PAT Group

The PAT plus standardized surgical consultation intervention was administered to patient-caregiver dyads. The PAT is a self-guided tablet-based interactive experience that combines a patient activation strategy with a decision aid for patient-caregiver dyads (https://vimeo.com/91207174) (eTable 2 and eTable 3 in Supplement 1). The PAT was designed to present understandable information about the treatments, increase the patient-caregiver dyad’s willingness to take an active role in their care, improve their confidence in their ability to manage the patient’s care, and provide them with the knowledge and skills necessary to make decisions.

### Study Outcomes

#### Primary Outcomes

This study had 3 primary outcomes: (1) caregiver decision self-efficacy immediately after the treatment decision using the Decision Self-efficacy Scale^18,19,20^; (2) caregiver health care satisfaction at discharge using the PedsQL (Pediatric Quality of Life Inventory) 4.0 Healthcare Satisfaction Generic Module–Parent Report^21,22^; and (3) disability days of the patient at 1 year. Disability days were defined as days in which the patient did not participate in their normal routine secondary to discomfort, symptoms, complications, or follow-up related to their appendicitis treatment.

#### Secondary Outcomes

Decision-making and patient-centered outcomes assessed in caregivers included decision self-efficacy at discharge and 30-day follow-up; preparation for decision making, decisional conflict, and Parent-Patient Activation Measure (Parent-PAM) immediately after treatment decision using the Preparation for Decision Making Scale,^19,23,24,25^ Decisional Conflict Scale,^26^ and a version of the healthy child Parent-PAM adapted with permission^27^; decision regret at discharge and 30-day follow-up using the Decision Regret Scale^28,29^; health-related quality of life at discharge and 30-day follow-up using self-reports and caregiver proxy reports on the PedsQL 4.0 Generic Core Scales–Child and Parent Report^30,31,32,33,34^; health care satisfaction at 30-day follow-up; satisfaction with decision at 30-day and 1-year follow-up, and caregiver knowledge or recall immediately after treatment decision and at 30-day follow-up using an internally created survey. Medical outcomes of the patient assessed included disability days, length of hospital stay, medical or surgical complications, recurrence, and readmissions. eTable 4 in Supplement 1 details all outcomes assessed. Complicated appendicitis was defined based on pathologic results and included ruptured, perforated, and gangrenous appendicitis.^16^

### Sample Size and Power

We expected the PAT to increase the total Decision Self-efficacy Scale score by 0.4 SD and increase the total caregiver PedsQL Healthcare Satisfaction Generic Module score at discharge by 0.5 SD.^35,36^ We expected the number of disability days in the standardized surgical consultation group to be 14 with an SD of 5,^37^ and we set our noninferiority margin to 14%, or 2 days more. We used the Holm procedure to test the 2 primary patient-centered outcomes at a 1-sided α = .02. This procedure was followed by a gatekeeping fallback procedure to test for noninferiority of disability days at the 1-sided α = .025 level if we achieved significance for 1 or both of the patient-centered outcomes or at the 1-sided α = .005 level if significance was not achieved on either.^38^ To control the overall type I error at α = .05, our significance level for the patient-centered primary outcomes was set at α = .02 (rather than α = .025). Based on this strategy, 100 patients in each group provided 92% power for either patient-centered outcome, 74% power for both, and 80% power to claim noninferiority for disability days.

### Statistical Analysis

Data were analyzed from May 1, 2014, to May 1, 2017. All randomized participants were included in the analyses. Baseline and descriptive variables are reported as medians with interquartile ranges (IQRs) where appropriate. We performed the preplanned hypothesis testing of the 2 primary patient-centered outcomes at a 1-sided α = .02 first. Because both were not significant, we followed the gatekeeping fallback procedure to test for noninferiority of disability days at the 1-sided α = .005 level. We also tested for superiority of disability days after the noninferiority test was failed.^39^ Owing to the nonsignificance of the primary outcomes, we only provided the summary statistics and tested decision-making and patient-reported outcomes between standardized consultation and PAT groups using nonparametric methods at each time point. All scored data were summarized and analyzed per the specification of each validated instrument. Secondary decision-making and patient-reported outcomes were compared between groups using 2-sided Wilcoxon rank sum tests. Medical outcomes were compared using 2-sided Pearson χ^2^ or Fisher exact tests. We compared rates of postoperative infections, reoperation, and readmission among patients undergoing surgery and rates of appendectomy among those undergoing nonoperative management using 2-sided Pearson χ^2^ or Fisher exact tests. For the primary outcomes, comparisons among the 4 subgroups based on the randomized intervention and treatment choice were performed using Kruskal-Wallis tests for continuous variables and Fisher exact tests for categorical variables. All analyses were conducted in SAS, version 9.4 (SAS Institute, Inc).

## Results

### Enrollment and Follow-up

The Figure details the study flow. From March 1, 2014, through April 30, 2016, 200 of 233 patients and their caregivers (31 declined to participate and 2 had no available guardian to consent) who met eligibility criteria were enrolled (85.8%). Of these 200 patients (120 [60.0%] male and 80 [40.0%] female; median age, 12 years [IQR, 9-15 years]), 98 (49.0%) were randomized to the PAT group and 102 (51.0%) were randomized to the standardized consultation group. Among the PAT patient-caregiver dyads, 74 (75.5%) received the PAT together, 2 (2.0%) received it separately, and 22 (22.4%) had only the caregiver receive it. No difference in the proportion of patients choosing nonoperative management or surgery occurred between the PAT group (antibiotics for 31 of 98 [31.6%] and surgery for 67 of 98 [68.4%]) and standardized consultation group (antibiotics for 42 of 102 [41.2%] and surgery for 60 of 102 [58.8%]; *P* = .19). No participant requested to speak with the surgical team before making their treatment decision, and no decisions changed after participants met the surgical team or attending physician. Baseline demographics and clinical characteristics in Table 1 demonstrate that the groups were similar in all measured variables. Caregivers in the 2 randomized groups had a significantly different distribution of responses when asked whether the information they received was biased (*P* = .01). Among the 76 participants assessed in the PAT group, a higher proportion reported bias toward antibiotics (64 [84.2%] reported no bias; 1 [1.3%], bias toward surgery; and 11 [14.5%], bias toward nonoperative management), whereas among the 77 assessed in the standardized consultation group, a higher proportion reported bias toward surgery (70 [90.9%] reported no bias; 5 [6.5%], bias toward surgery; and 2 [2.6%], bias toward nonoperative management).

**Figure.**
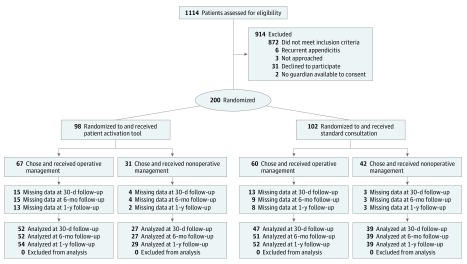
CONSORT Flow Diagram

**Table 1.** Baseline Demographics and Clinical Characteristics

Characteristic	Patient Group^a^		
	All (n = 200)	Standardized Consultation Group (n = 102)	PAT Group (n = 98)
Age, median (IQR), y	12 (9-15)	13 (9-15)	12 (10-14)
Male	120 (60.0)	61 (59.8)	59 (60.2)
White^b^	173 (86.5)	85 (83.3)	88 (89.8)
Primary language other than English	21 (10.5)	10 (9.8)	11 (11.2)
Insurance status			
Private	126 (63.0)	67 (65.7)	59 (60.2)
Medicaid	65 (32.5)	28 (27.5)	37 (37.8)
None	9 (4.5)	7 (6.9)	2 (2.0)
Highest level of parent/guardian education			
Less than a high school diploma/elementary	8 (4.0)	5 (4.9)	3 (3.1)
High school/GED	32 (16.0)	16 (15.7)	16 (16.3)
Some college	40 (20.0)	19 (18.6)	21 (21.4)
Associate degree	25 (12.5)	13 (12.7)	12 (12.2)
BS or BA degree	51 (25.5)	22 (21.6)	29 (29.6)
Master’s degree	30 (15.0)	19 (18.6)	11 (11.2)
PhD degree	1 (0.5)	1 (1.0)	0
Professional	5 (2.5)	3 (2.9)	2 (2.0)
Not documented	8 (4.0)	4 (3.9)	4 (4.1)
Total household income, $			
<25 000	25 (12.5)	15 (14.7)	10 (10.2)
25 000-49 999	42 (21.0)	22 (21.6)	20 (20.4)
50 000-99 999	49 (24.5)	22 (21.6)	27 (27.6)
≥100 000	72 (36.0)	38 (37.3)	34 (34.7)
Not documented	12 (6.0)	5 (4.9)	7 (7.1)
Single or double income			
Single	84 (42.0)	46 (45.1)	38 (38.8)
Double	83 (41.5)	40 (39.2)	43 (43.9)
Not documented	33 (16.5)	16 (15.7)	17 (17.3)
Parent/guardian marital status			
Never married	20 (10.0)	9 (8.8)	11 (11.2)
Married	115 (57.5)	58 (56.9)	57 (58.2)
Separated	4 (2.0)	3 (2.9)	1 (1.0)
Divorced	25 (12.5)	13 (12.7)	12 (12.2)
Widowed	3 (1.5)	3 (2.9)	0
Not documented	33 (16.5)	16 (15.7)	17 (17.3)
Presenting symptoms			
Fever	41 (20.5)	21 (20.6)	20 (20.4)
Vomiting	86 (43.0)	45 (44.1)	41 (41.8)
Diarrhea	19 (9.5)	9 (8.8)	10 (10.2)
Imaging at presentation			
Ultrasonography	158 (79.0)	79 (77.5)	79 (80.6)
Computed tomography	56 (28.0)	27 (26.5)	29 (29.6)
White blood cell count, median (IQR), /μL	11.8 (9.6-14.6)	11.8 (9.0-14.5)	11.7 (9.8-15.0)
Duration of abdominal pain at presentation to ED, median (IQR), h	18 (12-24)	18 (12-24)	17 (12-24)
Hospital length of stay, median (IQR), h	23 (16-32)	24 (17-34)	21 (16-30)

Abbreviations: ED, emergency department; GED, General Educational Development; IQR, interquartile range; PAT, patient activation tool.

SI conversion factor: To convert white blood cell count to ×10^9^ per liter, multiply by 0.001.

^a^ Unless otherwise indicated, data are expressed as number (percentage) of patients.

^b^ Self-reported by caregiver from prespecified categories.

### Primary Outcomes

Decisional self-efficacy within 1 hour was high in the standardized consultation (among 101 dyads assessed) and PAT (among 95 dyads assessed) groups (median score, 100 [IQR, 97.7-100] vs 100 [IQR, 95.5-100]; *P* = .03; not significant at planned significance level of *P* = .02). The median scores of 100 indicated extremely high self-efficacy based on this scale. Caregiver health care satisfaction at discharge was similarly high in both groups, with no significant difference between the standardized consultation (among 100 dyads assessed) or PAT (among 94 dyads assessed) groups (median score, 99.0 [IQR, 94.7-100] vs 98.0 [IQR, 91.7-100]; *P* = .27). Disability days at 1 year were also similar between the standardized consultation (among 91 assessed) and PAT (among 83 assessed) groups (median score, 6 [IQR, 2-11] vs 5 [IQR, 2-15]; *P* = .67). The noninferiority test of disability days was also not significant (*P* = .07).

When we examined the primary outcomes based on randomized group and treatment choice, we found a significant difference in decisional self-efficacy, with patients in the PAT group who chose antibiotics having the lowest median score (97.7 [IQR, 93.2-100] compared with median scores of 100 for the standardized consultation group choosing surgery [IQR, 100-100], the PAT group choosing surgery [IQR, 95.5-100], and the standardized consultation group choosing antibiotics [IQR, 95.5-100]; *P* = .02). The high median scores demonstrate extremely high self-efficacy, suggesting the statistical difference may not be clinically meaningful. We found no significant differences based on the randomized group and treatment choice in median disability days at 1 year (standardized consultation group choosing surgery, 8 days [IQR, 2-15 days]; standardized consultation group choosing antibiotics, 4 days [IQR, 2-7 days]; PAT group choosing surgery, 8 days [IQR, 3-15 days]; PAT group choosing antibiotics, 3 days [IQR, 1-16 days]; *P* = .10) or median health care satisfaction score at discharge (standardized consultation group choosing surgery, 99.0 [IQR, 89.6-100]; standardized consultation group choosing antibiotics, 99.0 [IQR, 95.9-100]; PAT group choosing surgery, 97.4 [IQR, 87.5-100]; PAT group choosing antibiotics, 93.9 [IQR, 76.1-100]; *P* = .35).

### Decision-Making and Patient-Centered Secondary Outcomes

Decisional self-efficacy scores were similar between the groups at discharge and 30-day follow-up (Table 2). The distribution of scores on the Preparation for Decision Making Scale were significantly different in the PAT group compared with the standardized consultation group immediately after the decision (100 [IQR, 80.0-100] vs 100 [IQR, 87.5-100]; *P* = .03) (Table 2). The high median scores reflect being a great deal prepared for decision making, suggesting that the statistical difference is not likely clinically meaningful. No significant differences were found on measures of caregiver-patient activation or decisional conflict immediately after the decision or in health-related quality of life, health care satisfaction, decisional regret, satisfaction with decision, or guardian knowledge or recall at any point (Table 2).

**Table 2.** Decision-Making and Patient-Reported Outcomes

Outcome Measured by Time	No. of Patients Assessed	Median (IQR)		*P* Value
		Standardized Surgical Consultation Group	PAT Group	
Decision Self-efficacy Scale score^a^				
At discharge	193	100 (97.7-100)	100 (97.7-100)	.41
30 d	150	100 (95.5-100)	100 (90.5-100)	.38
Preparation for Decision Making Scale score^b^				
Immediately after decision	197	100 (87.5-100)	100 (80.0-100)	.03
Parent-Patient Activation Measure score^c^				
Immediately after decision	196	100 (77.7-100)	100 (75.0-100)	.17
Health care satisfaction score during initial hospitalization^d^				
30 d	150	99.0 (92.8-100)	96.9 (84.5-100)	.18
PedsQL 4.0 Generic Core Scales score, parent reported^e^				
At discharge	193	89.1 (81.5-94.6)	89.1 (77.2-94.6)	.39
30 d	147	88.0 (81.0-96.7)	88.0 (79.3-96.7)	.99
1 y	164	93.5 (85.3-98.9)	92.9 (83.2-98.9)	.49
PedsQL 4.0 Generic Core Scales score, parent reported for patient^e^				
At discharge	192	90.2 (82.6-94.6)	89.7 (78.3-96.7)	.71
30 d	149	90.2 (80.4-97.8)	87.0 (77.2-94.6)	.21
1 y	169	94.6 (87.0-100)	92.4 (85.9-97.8)	.25
Decisional Conflict Scale score^f^				
Immediately after decision	197	0 (0-0)	0 (0-0)	.42
Decision Regret Scale score^g^				
At discharge	192	40 (40-45)	40 (40-45)	.32
30 d	163	40 (40-45)	40 (40-40)	.58
Guardian Knowledge Recall Assessment, % correct^h^				
Immediately after decision	200	100 (100-100)	100 (100-100)	.18
30 d	163	100 (100-100)	100 (100-100)	.11
Satisfaction with decision score^d^				
30 d	165	100 (83.3-100)	100 (80-100)	.84
1 y	174	100 (96.7-100)	100 (93.3-100)	.20

Abbreviations: IQR, interquartile range; PAT, patient activation tool; PedsQL, Pediatric Quality of Life Inventory.

^a^ Scores range from 0 to 100, with higher scores indicating more confidence.

^b^ Scores range from 0 to 100, with higher scores indicating higher perceived preparation.

^c^ Scores range from 0 to 100, with higher scores indicating higher activation.

^d^ Scores range from 0 to 100, with higher scores indicating greater satisfaction.

^e^ Scores range from 0 to 100, with higher scores indicating higher health-related quality of life.

^f^ Scores range from 0 to 100, with higher scores indicating more conflict.

^g^ Scores range from 0 to 100, with higher scores indicating greater regret.

^h^ Scores range from 0 to 100, with higher scores indicating better recall.

### Secondary Medical Outcomes

At 30 days and 1 year after discharge, no significant differences occurred in any of the measured medical outcomes, including rates of complicated appendicitis, hospital readmission, emergency department or urgent care visits, postoperative complications, or disability days (Table 3). Among patients who received nonoperative management, there was no difference in the treatment failure rate of nonoperative management or in the rate of complicated appendicitis among those in whom nonoperative management failed (Table 3).

**Table 3.** Medical Outcomes

Patient-Reported Outcome	Patient Group^a^		Relative Risk (95% CI)	*P* Value
	Standardized Consultation Group	PAT Group		
**30 d After Discharge**				
Complicated appendicitis	7/68 (10.3)	9/71 (12.7)	0.70 (0.24-2.02)	.79
Hospital readmission	8/87 (9.2)	7/79 (8.9)	1.04 (0.39-2.73)	>.99
ED or UC visit	4/86 (4.7)	5/79 (6.3)	0.73 (0.20-2.64)	.74
Postoperative wound infection	1/54 (1.9)	0/53	Undefined	>.99
Disability days, median (IQR)	5.5 (2-11)	5 (2-10)	NA	.68
School days missed, median (IQR)	2 (1-4)	2 (1-4)	NA	.60
Normal activity days missed, median (IQR)	4.5 (1-10)	3.5 (1-9)	NA	.79
Guardian days missed from normal activities, median (IQR)	2 (1-4)	2 (1-4)	NA	.93
Failure of nonoperative management or recurrence of appendicitis				
Any	8/42 (19.0)	4/30 (13.3)	1.43 (0.47-4.31)	.75
Complicated	2/42 (4.8)	1/30 (3.3)	1.43 (0.14-15.04)	>.99
**1 y After Discharge**				
Hospital readmission	15/92 (16.3)	15/83 (18.1)	0.90 (0.47-1.73)	.84
ED or UC visit	6/91 (6.6)	6/83 (7.2)	0.91 (0.31-2.72)	>.99
Disability days, median (IQR)	6 (2-11)	5 (2-15)	NA	.67
School days missed, median (IQR)	2 (1-4)	3 (1-5)	NA	.36
Normal activity days missed, median (IQR)	5 (1-10)	5.5 (1-14)	NA	.76
Guardian days missed from normal activities, median (IQR)	2 (1-4)	2 (1-4)	NA	.43
Failure of nonoperative management or recurrence of appendicitis				
Any	13/38 (34.2)	11/30 (36.7)	0.93 (0.49-1.78)	>.99
Complicated	2/38 (5.3)	1/30 (3.3)	1.58 (0.15-16.59)	>.99

Abbreviations: ED, emergency department; IQR, interquartile range; NA, not applicable; PAT, patient activation tool; UC, urgent care.

^a^ Unless otherwise indicated, data are expressed as number/total number (percentage) of patients assessed.

### Secondary Analyses Based on Treatment Choice

Demographic, socioeconomic, and clinical characteristics were similar between patients choosing surgery and those choosing nonoperative management except that patients who chose nonoperative management were less likely to be white (56 of 73 [76.7%] vs 117 of 127 [92.1%]; *P* = .004) and more likely to have a primary language other than English (14 of 73 [19.2%] vs 7 of 127 [5.5%]; *P* = .004). The 1-year success rate of nonoperative management was 64.7% (44 of 68). Overall rates of complicated appendicitis were 4.4% (3 of 68) in patients choosing nonoperative management and 10.2% (13 of 127) in those choosing surgery. Differences in outcomes based on choosing surgery vs nonoperative management are shown in Table 4. For the primary outcomes, no significant difference occurred in decisional self-efficacy immediately after the decision or in health care satisfaction at discharge. At 1-year follow-up, patients choosing nonoperative management had significantly fewer disability days (median, 3 [IQR, 1-8]) compared with patients choosing surgery (median, 3 [IQR, 2-15]; *P* < .001). Quality-of-life scores at 30-day follow-up were higher in patients choosing nonoperative management (median, 92.4 [IQR, 79.3-100] vs 87.0 [IQR, 78.3-93.5]; *P* = .01). No clinically significant differences occurred in the decisional conflict, decision regret, quality of life, or satisfaction with the decision scores between dyads choosing surgery and those choosing nonoperative management.

**Table 4.** Outcomes for Secondary Analyses Based on Treatment Choice

Outcome Measured	No. of Patients	Treatment Choice^a^		*P* Value
		Surgery	Nonoperative Management	
Decision Self-efficacy Scale score^b^				
Immediately after decision	196	100 (97.7-100)	100 (93.2-100)	.051
Health care satisfaction score during hospitalization^c^				
At discharge	194	98.9 (94.3-100)	97.8 (92.4-100)	.17
PedsQL 4.0 Generic Core Scales score, parent reported^d^				
At discharge	193	89.1 (80.4-94.6)	87.0 (77.2-93.5)	.23
30 d after discharge	147	88.0 (79.3-94.6)	91.8 (80.4-100)	.07
1 y after discharge	164	93.5 (83.7-100)	92.4 (84.8-98.9)	.73
PedsQL 4.0 Generic Core Scales score, parent reported for patient^d^				
At discharge	192	89.1 (81.5-95.7)	91.3 (80.4-96.7)	.94
30 d after discharge	149	87.0 (78.3-93.5)	92.4 (79.3-100)	.01
1 y after discharge	169	94.6 (84.8-98.9)	93.5 (88.0-100)	.51
Decisional Conflict Scale score^e^				
Immediately after decision	197	0 (0-0)	0 (0-0)	.01
Decision Regret Scale score^f^				
At discharge	192	40 (40-40)	40 (40-45)	.14
30 d after discharge	163	40 (40-40)	40 (40-45)	.001
Satisfaction with decision score^c^				
30 d after discharge	165	100 (86.7-100)	100 (80-100)	.17
1 y after discharge	174	100 (100-100)	100 (83.3-100)	.01
Complicated appendicitis, No./total No. (%)				
30 d after discharge	127	13/127 (10.2)	NA	NA
Hospital readmission, No./total No. (%)				
30 d after discharge	166	2/99 (2.0)	13/67 (19.4)	<.001
1 y after discharge	175	2/106 (1.9)	28/69 (40.6)	<.001
ED or UC visit, No./total No. (%)				
30 d after discharge	165	3/99 (3.0)	6/66 (9.1)	.16
1 y after discharge	174	3/106 (2.8)	9/68 (13.2)	.01
Postoperative wound infection, No./total No. (%)				
30 d after discharge	99	1/99 (1.0)	NA	NA
Disability days				
30 d after discharge	165	8 (4-15)	2 (1-5)	<.001
1 y after discharge	174	3 (2-15)	3 (1-8)	.01
School days missed				
30 d after discharge	165	3 (2-5)	1 (0-3)	<.001
1 y after discharge	174	3 (2-5)	2 (0-4)	.02
Normal activity days missed				
30 d after discharge	165	7 (3-14)	1 (0-4)	<.001
1 y after discharge	174	7 (3-14)	2 (0-6)	<.001
Guardian days missed from normal activities				
30 d after discharge	165	3 (1-4)	2 (1-3)	.16
1 y after discharge	174	2 (1-4)	2 (1-5)	.72
Failure of nonoperative management or recurrence of appendicitis, No./total No. (%)				
Any				
30 d after discharge	72	NA	12/72 (16.7)	NA
1 y after discharge	68	NA	24/68 (35.3)	NA
With complicated appendicitis				
30 d after discharge	72	NA	3/72 (4.2)	NA
1 y after discharge	68	NA	3/68 (4.4)	NA

Abbreviations: ED, emergency department; NA, not applicable; PedsQL, Pediatric Quality of Life Inventory; UC, urgent care.

^a^ Unless otherwise indicated, data are expressed as median (interquartile range).

^b^ Scores range from 0 to 100, with higher scores indicating more confidence.

^c^ Scores range from 0 to 100, with higher scores indicating greater satisfaction.

^d^ Scores range from 0 to 100, with higher scores indicating higher health-related quality of life.

^e^ Scores range from 0 to 100, with higher scores indicating more conflict.

^f^ Scores range from 0 to 100, with higher scores indicating greater regret.

### Post Hoc Secondary Analyses: Successful vs Failed Nonoperative Management

Among patients choosing nonoperative management (n = 73), no significant differences occurred in demographic, socioeconomic, and clinical characteristics between successful (n = 48) and failed (n = 25) nonoperative management. We found no significant differences in any patient-reported outcome, including the primary outcomes of decisional self-efficacy and health care satisfaction (eTable 5 in Supplement 1). Satisfaction with the decision scores were similar for those who had successful nonoperative management and those who had failed nonoperative management at the 30-day (median score, 100 [IQR, 80.0-100] vs 88.3 [IQR, 45.0-100]; *P* = .19) and 1-year (median score, 100 [IQR, 80.0-100] vs 100 [IQR, 80.0-100]; *P* = .39) follow-up (eTable 5 in Supplement 1).

## Discussion

The goal of this study was to determine whether a PAT can be successfully used in an acute care setting to activate pediatric patient-caregiver dyads and improve decision-making and patient-centered outcomes without compromising medical outcomes. We did not detect any clinically meaningful differences between the PAT and standardized surgical consultation interventions alone in measures of decision making, patient-centered outcomes, or medical outcomes. However, we found that both groups had high scores on tests that measure caregiver ability and comfort with making an acute treatment decision between surgery and antibiotics alone for their child’s appendicitis. This finding suggests that patients and families could process information about the risks and benefits of treatment options in the acute-care setting and effectively participate in an informed decision-making process with the medical team.

Incorporating the perspectives of patients and families into clinical practice and promoting shared decision making are becoming priorities throughout medical care. Our results may help inform these efforts. Both randomized groups had high scores on scales assessing decisional self-efficacy, preparation for decision making, caregiver activation, health care satisfaction, and satisfaction with decision. These results suggest that both groups were well informed and well prepared to participate in treatment decision making. They demonstrate that consultations that encourage patient participation (verbal or technology based) in the emergency and urgent care settings can lead to high scores on measures of decision making.

The call for shared decision making to be a part of medical care began when the Institute of Medicine report *Crossing the Quality Chasm: A New Health System for the 21st Century* defined patient-centered care as “care that is respectful of and responsive to individual patient preferences, needs, and values” and ensures “that patient values guide all clinical decisions.”^40^^(p40)^ Shared decision making involves physicians and patients working together to make treatment decisions that balance the risks and expected outcomes with patient preferences and values.^1^ Shared decision making should occur when more than 1 reasonable treatment option is available, no single option has a clear advantage, and the risks and benefits of each option affect patients differently. Shared decision making has traditionally not been a part of the treatment of uncomplicated appendicitis because surgery had long been considered the only reasonable treatment.^1^ However, in the last 15 years, several clinical trials and meta-analyses^16,17,41,42,43,44,45,46,47,48,49,50,51,52,53,54,55,56,57,58,59,60^ have established the safety and efficacy of treating uncomplicated appendicitis nonoperatively with antibiotics alone. The currently available data support surgery and antibiotics alone as reasonable treatments for uncomplicated appendicitis that should be offered as part of a shared decision-making process.^61,62,63^ Each treatment has distinct advantages based on how a patient values minimizing the risk of recurrence or avoiding surgery. Surgery is an invasive procedure with associated disability, pain, and risks of complications, whereas nonoperative management is noninvasive but comes with the risk of failure or recurrence that may eventually need surgery. Although our study did not demonstrate a benefit of the PAT, the overall high scores on measures of decision making suggest that children and their caregivers can successfully participate in shared decision making in the acute-care setting and make an informed treatment decision for their child’s appendicitis.

Most of the studies on patient activation, decision making, and patient-centered outcomes focus on autonomous adult patients and on chronic illness and/or outpatient care.^64,65,66,67,68^ In adults, patient activation has been shown to improve healthy behaviors, increase the appropriate use of health care resources, improve patient preparedness for medical appointments, and improve the management and control of chronic illnesses such as diabetes.^3,4,5,6,7,8,69^ Several studies have demonstrated that decision aids can improve patient knowledge, a component of activation, in patients undergoing major elective surgery.^64^ However, none of these studies targeted acute surgical procedures or children. This study specifically investigated the effects of patient activation on decision-making and patient-centered outcomes in an acute, often surgically treated disease in children. To date, and to our knowledge, this study is the first to combine a patient activation strategy with a decision aid for patient-caregiver dyads in the acute surgical setting. This study did not find a difference in measures of decision making, patient-centered outcomes, or medical outcomes between a PAT, designed to activate caregivers in the emergency setting through knowledge and engagement, and a standardized consultation that emphasized patient choice. However, this study demonstrated extremely high scores on measures of decision making in both groups, which may reflect a ceiling effect of the intervention.

The failure rate of nonoperative management in this study was consistent with that in previous trials^44,47,49^ but higher than in a previous study investigating nonoperative management at a single institution.^16^ The shared decision-making process may have led several families who would have chosen surgery to attempt initial nonoperative management. Consequently, this choice may have contributed to the higher failure rate. In addition, the higher failure rate may be due, in part, to the focus of this trial on investigating decision making rather than comparing these 2 treatments. As such, compliance with established treatment protocols for nonoperative management after enrollment was left to the clinical team caring for the patient and not subject to rigorous research monitoring and enforcement. This situation may have allowed for increased crossover to appendectomy based on subjective criteria. This possibility highlights the potential that the effectiveness of nonoperative management in clinical practice may be different than estimates reported in rigorously controlled clinical trials.

### Limitations

This study has several limitations. First, it is a single-site trial performed at a tertiary children’s hospital in the Midwest, and therefore the results may not be generalizable. However, our institution is the primary surgical facility for children from urban and rural communities, and this trial had broad enrollment, with 85.8% of approached eligible patients enrolling. Second, the study was single-blind, which may introduce bias; attempts to minimize this bias include having assessors of outcomes at or after discharge blind to group assignment. Third, we compared the PAT with a standardized scripted surgical consultation that emphasized the importance of patient values and participation in their care. This standardized consultation may have activated and encouraged patients and their families to participate in the treatment decision-making process more than a typical consultation in clinical practice, which may have limited our ability to detect a difference by increasing measures of decision making in the control group. The choice to not use a usual care comparator group was based on the Patient-Centered Outcomes Research Institute guidelines to avoid usual care comparators and our stakeholders’ input that the control group should be standardized and represent the best available standard of care. Also, it is possible that developing and testing a tool similar to the PAT but in a different acute disease process could demonstrate benefit.

## Conclusions

The results of this study suggest that a consultation that promotes shared decision making may lead to confident caregiver decision making in the acute care setting. Our technology-based tool did not improve measures of decision making compared with a standardized consultation that emphasized patient choice. However, both groups had high scores, suggesting that caregivers in both groups were well informed and well prepared to participate in their child’s treatment decision. This finding challenges the widely held notion that families of pediatric patients are generally unable or ill-equipped to participate in shared decision-making efforts around acute, invasive treatment choices. Additional research is needed to determine whether providing a comprehensive consultation that promotes shared decision making can lead to improved patient and family involvement in medical care in the acute care setting compared with usual care alone.

## Notes

Supplement 1. **eTable 1.** Summary of Stakeholder Team, Engagement and Contributions **eTable 2.** Comparison of Content Presented in the Standardized Surgical Consultation and PAT **eTable 3.** Content of Interventions **eTable 4.** Study Outcomes Assessed **eTable 5.** Outcomes for Post Hoc Secondary Analyses of Patients Choosing Nonoperative Management Based on Success vs Failure of Nonoperative Management **eReferences.**

Supplement 2. **Trial Protocol**

Supplement 3. **Data Sharing Statement**
