# Supplementary material for: Effects of a Patient Activation Tool on Decision Making Between Surgery and Nonoperative Management for Pediatric Appendicitis: A Randomized Clinical Trial
Source: JAMA Netw Open. 2019 Jun 7;2(6):e195009. doi: 10.1001/jamanetworkopen.2019.5009 (PMC6563561; doi:10.1001/jamanetworkopen.2019.5009)
Supplement: Supplement 1. — eTable 1. Summary of Stakeholder Team, Engagement and Contributions eTable 2. Comparison of Content Presented in the Standardized Surgical Consultation and PAT eTable 3. Content of Interventions eTable 4. Study Outcomes Assessed eTable 5. Outcomes for Post Hoc Secondary Analyses of Patients Choosing Nonoperative Management Based on Success vs Failure of Nonoperative Management eReferences. [file jamanetwopen-2-e195009-s001.pdf]

Supplementary Online Content

Minneci PC, Cooper JN, Leonhart K, et al. Effects of a patient activation tool on decision making between surgery and nonoperative management for pediatric appendicitis: a randomized clinical trial. *JAMA Netw Open.* 2019;2(6):e195009. doi:10.1001/jamanetworkopen.2019.5009

eTable 1. Summary of Stakeholder Team, Engagement and Contributions

eTable 2. Comparison of Content Presented in the Standardized Surgical Consultation and PAT

eTable 3. Content of Interventions

eTable 4. Study Outcomes Assessed

eTable 5. Outcomes for Post Hoc Secondary Analyses of Patients Choosing Nonoperative Management Based on Success vs Failure of Nonoperative Management

eReferences.

This supplementary material has been provided by the authors to give readers additional information about their work.

© 2019 Minneci PC et al. *JAMA Network Open*

eTable 1. Summary of Stakeholder Team, Engagement and Contributions

Stakeholder Team Members:

- 6 Patients with appendicitis and their families
- 1 Surgery clinic nurse
- 1 Child-family educator
- 1 Emergency Medicine physician
- 1 Community pediatrician
- 1 Payer representative

Stakeholder Engagement Process:

Stakeholder engagement during initial 6 months:

Initial stakeholder involvement during the first 6 months of this project included several rounds of individual interviews, in-person group meetings, phone calls, and email communications to develop the research question, design the study, develop the intervention, and choose the outcomes for this project After completing the initial interviews, we held a group meeting to review the feedback, the proposed study question, and the study design. We then began an iterative process with the stakeholders to develop the patient activation tool (PAT). We started by developing lists of the potential risks and benefits of each treatment option. These lists were reviewed with the stakeholders by email to ensure completeness and comprehension. We created paper versions of these lists and developed scripts that explained the two treatment options and their risks and benefits. At our second in-person group meeting, we reviewed this content and had an open discussion about the format for the PAT. The research team then worked with a film company and a software company to develop a prototype PAT that could be viewed and used by both the patients and their caregivers. This was reviewed with each stakeholder individually prior to our next group meeting in which we reviewed the feedback and came to consensus for additional changes.

Stakeholder engagement after 6 months:

After the first 6 months of the study, stakeholders were engaged at least 4 times per year with semi-annual, in-person group meetings and quarterly phone/email communications. Following each semi-annual meeting, study updates were sent to communicate the final actions to be implemented on issues discussed during the meeting. Quarterly email/phone updates were used to: (1) inform stakeholders on the progress of the study, (2) communicate any results that emerged due to stakeholder input, and (3) solicit questions and suggestions to increase the study's success.

**Stakeholder Team Contributions to the Project:** *Defining the decisional context:*
• Identified that decision-making between an appendectomy or antibiotics alone was the hardest part of the process
• Recognized that it is difficult to take the necessary time to fully explain the risks and benefits of each choice within the time constraints of an encounter in the Emergency Department
• Determined that there was a role to further study methods for improving shared decision-making.
Developing study question and design:
• Selected randomized controlled design to compare a standardized consultation to a patient activation tool (PAT)
• Determined that the goals of the intervention should be to engage, educate, and activate patients and their caregivers
• Favored a tablet-based application as the intervention
• Suggested inclusion of a patient activation module to facilitate engagement
• Selected the primary and secondary patient-centered outcomes
Development of the PAT
• Decided that an interactive tablet-based PAT would be the most engaging to both children and caregivers
• Selected lists of the potential risks and benefits of each treatment option

© 2019 Minneci PC et al. *JAMA Network Open*

• Specific content based on consensus of stakeholder input:
○ Inclusion of multiple different avatars that were sensitive to skin color, gender, and age
○ Time limitation of < 20 minutes for the length of the experience
○ Additional vignettes from patients and caregivers
○ Specific visual format for how the treatments were described
○ 2 x 2 grid format comparing the risks and benefits of each treatment
○ Graphics that assisted with low numeracy levels
○ Specifics of the interactive values exercise that helps patients and caregivers prioritize the risks and benefits for themselves
○ Inclusion of an optional module that provides additional training on techniques for communicating with healthcare providers
Development of the standardized consultation
• Suggested that it should represent best available standard of care<br>• Recommended that it minimize variation in how treatment options are explained across surgeons
• Specific content based on consensus of stakeholder input:
○ Explanation of the risks and benefits of each treatment choice
○ Explanation of the importance of patient-caregiver preferences and values when making medical decisions
Trial logistics
• Revised enrollment process to have a physician perform the enrollment
• Changed recruitment script
• Changed follow-up process

© 2019 Minneci PC et al. *JAMA Network Open*

eTable 2. Comparison of Content Presented in the Standardized Surgical Consultation and PAT

	Standardized surgical consultation	PAT +Standardized surgical consultation
Explained treatment options verbally	X	X
Explained risks and benefits verbally	X	X
Explained importance of patient-caregiver preferences and values when making a treatment decision	X	X
Opportunity to ask questions	X	X
Video explanation of treatments		X
Video explanation of risks and benefits		X
Summarized side by side comparison of risks and benefits		X
Graphics that visually summarized risk and benefits (numeracy)		X
Patient and caregiver vignettes discussing most common concerns about treatment		X
Patient and caregiver vignettes discussing most common reasons for choosing each treatment		X
Interactive values exercise to assist patients and caregivers in prioritizing the risks and benefits for themselves		X
Training on the importance of and techniques for communicating with healthcare providers		X
Embedded activation and communication strategies		X

© 2019 Minneci PC et al. *JAMA Network Open*

eTable 3. Content of Interventions

**Standardized surgical consultation script:**
Appendicitis is inflammation/infection of your appendix.
Since your child's appendicitis was diagnosed early, there are two treatment options: surgery or non-operative treatment with antibiotics alone. These two treatment options are both reasonable choices but they have very different risks and benefits. We will review what each treatment involves and then the associated risks and benefits of each.
The first option is an appendectomy which is surgery to remove your child's appendix. If you choose this option, your child will be admitted and given IV antibiotics up until surgery. For surgery, your child will be given general anesthesia to put him/her to sleep. Surgery is performed by making three small incisions and using a camera and two instruments to find and remove the appendix. The incisions are closed and your child is awoken from anesthesia. The entire procedure takes about one hour. After surgery, patients usually stay in the hospital one to two days. Your child can usually return to school in few days and resume all activities, including sports, in about two weeks.
The second option is to treat your child's appendicitis with antibiotics alone. If you choose this option, your child will be admitted and given IV antibiotics for at least one day. Usually, with antibiotics alone, patients stay in the hospital one to two days. Doctors and nurses will check on your child frequently to make sure he/she is getting better. When your child feels better and is able to eat, he or she will continue taking the antibiotics by mouth for about 7 days at home. Children can typically return to all activities, including school and sports, in two to three days.
Either of these treatment options is reasonable and there is no right or wrong choice. However, there is likely one choice that is best for you and your child depending on which of the risks and benefits are most important to you.
In order to help you make the best decision for your child and family, let's look at the risks and benefits of each treatment option.
The benefits of non-operative treatment with antibiotics alone include:
- Antibiotics alone have been shown to be a safe method to treat children with acute appendicitis. About 8 of 10 children never need surgery
- Your child's pain may go away faster and he/she will recover sooner.
- If your child never needs surgery, then there are no risks of surgery.
The possible risks of non-operative treatment may include:
- Your child's symptoms might not go away and he/she will need an appendectomy which involves the risks of surgery. About 1 of 10 children do not get better and will need surgery while in the hospital
- Your child's appendicitis could come back in the future. About 1 out of 10 children will have appendicitis again.
- Altogether, about 2 out 10 patients treated with antibiotics alone will eventually need an appendectomy.
- There can be side effects of antibiotics
o Most common: nausea, vomiting and diarrhea
The benefits of surgery include:
- Surgery is curative. Your child will never have appendicitis again.
- Surgery is the most common way to treat appendicitis. About 9 out of 10 children will not have a complication after appendectomy.
- Your child can usually go home within 1 to 2 days after surgery
The possible risks of surgery may include:
- Your child will be in some pain after surgery

- Most kids need a few days of rest before going back to school and 1-2 weeks before resuming physical activity
- It will leave 1-3 small scars on your belly
- There are some risks during surgery, such as bleeding or problems from the anesthesia. About 1 out of 10 patients experience a complication.
- The most common complications are minor and include infections or problems with the wounds.
- Other possible risks also include:
o Developing an abdominal abscess (an infection inside your child's belly)
o Scars (adhesions) that can cause future blockage in your child's belly that may require additional surgery
- If your child has a complication, then his/her hospital stay could be extended and he/she may need more medications such as antibiotics
Both surgery and non-operative treatment with antibiotics alone are good treatments for your child's appendicitis. Either of these treatment options is reasonable and there is no right or wrong choice. You should choose whichever treatment is best for your child and family based on which risks and benefits are most important to you.
**Patient activation tool (PAT) design and content:** The PAT was developed based on the concept of the patient activation continuum and the potential for improved outcomes in more activated patients compared to less activated patients.[2] The definition of activation used stemmed from a combination of the conceptual model proposed by Hibbard et al. and the Ottawa Decision Support Framework.[1,2] We defined an activated patient-caregiver dyad primarily on the willingness, knowledge, engagement, and self-efficacy of the caregiver. Consequently, we developed an integrated PAT that activates the patient-caregiver dyad, provides knowledge and skills regarding the medical decision, and strengthens self-efficacy in order to assist the caregiver in his/her treatment choice. Compared to the scripted surgical consultation, the PAT provided additional content such as: video explanations of the treatments and their risks and benefits; vignettes of caregivers and children explaining the most commons reasons for choosing each treatment; an interactive exercise to help caregivers and patients align their preferences with their treatment choice; and embedded activation and communication strategies based on components of the PACE (Presenting, Asking, Checking, Expressing) system.[3-6] The PAT was designed to be used by both caregivers and patients and was tailored to a Flesch-Kincaid reading level between 5th and 6th grade.<br><br>A video demonstration of the PAT can be found at https://vimeo.com/91207174

© 2019 Minneci PC et al. *JAMA Network Open*

eTable 4. Study Outcomes Assessed

Measured Outcomes	Measurement Instrument	Person Reporting	Time Points					
			Index Admission	Immediately	At Discharge	30 Days	6 Months	1 Year
**In All Patients**								
Length of stay	Medical Chart	Medical Chart	X					
Decisional self-efficacy and confidence	Decisional Self-Efficacy Scale	Caregiver			X	X		
Preparedness for decision-making	Preparation for Decision-making Scale[5]	Caregiver		X				
Caregiver activation level	Parent Patient Activation Measure®	Caregiver		X				
Healthcare Satisfaction during the initial hospitalization	PedsQL™ 3.0 Healthcare Satisfaction Generic Module	Caregiver			X	X		
Quality of life (QOL)	PedsQL™ 4.0 Generic Core Scales	Patient and Caregiver			X	X		
Certainty with treatment choice	Decisional Conflict Scale	Caregiver		X				
Recall (knowledge) about the disease and treatment options	Study Specific Knowledge Survey	Caregiver		X	X			
Remorse or Regret with treatment choice	Decision Regret Scale	Caregiver			X	X		
Satisfaction with Decision	Satisfaction with Decision Scale	Caregiver				X		X
Disability days	Study Specific Surveys	Patient and Caregiver				X	X	X
Readmissions	Study Specific Surveys/Chart Review	Medical Chart Caregiver				X	X	X
**In Operative Patients Only**								
Postoperative infections	Study Specific Surveys/Chart Review	Medical Chart Caregiver				X		
Re-operation	Study Specific Surveys/Chart Review	Medical Chart Caregiver				X		X

© 2019 Minneci PC et al. *JAMA Network Open*

Readmissions	Study Specific Surveys/Chart Review	Medical Chart Caregiver				X	X	X
**<u>In Non-Operative Patients Only</u>**								
Need for appendectomy during initial admission	Study Specific Surveys/Chart Review	Medical Chart	X					
Recurrence	Study Specific Surveys/Chart Review	Medical Chart Caregiver				X		X
Antibiotic complications	Study Specific Surveys/Chart Review	Medical Chart Caregiver				X		

© 2019 Minneci PC et al. *JAMA Network Open*

eTable 5. Outcomes for Post Hoc Secondary Analyses of Patients Choosing Nonoperative Management Based on Success vs Failure of Nonoperative Management

Outcome	Time Point	N	Successful Non-operative<br>N/total N (%) or median [IQR]	Failed Non-operative<br>N/total N (%) or median [IQR]	P-value
Decisional self-efficacy scales<br>scores 0-100; higher scores indicate more confidence	Immediately after decision	71	100 [93.2-100]	100 [95.5-100]	0.94
	At discharge	71	100 [97.7-100]	100 [92-100]	0.40
	30 days post-discharge	59	100 [90.9-100]	98.9 94.3-100]	0.83
Preparation for decision making scale	Immediately after decision	71	96.3 [85-100]	97.5 [90-100]	0.27
scores 0-100; higher scores indicate higher perceived preparation					
Parent patient activation measure	Immediately after decision	71	84.8 [75-100]	77.7 [75-100]	0.39
scores 0-100; higher scores indicate higher activation					
Healthcare satisfaction during hospitalization	30 days post-discharge	59	98 [81.4-100]	96.9 [91.7-100]	0.8
scores 0-100; higher scores indicate higher satisfaction					
Quality of Life- Parent Reported	At discharge	70	89.1 [77.2-96.7]	91.8 [83.7-96.7]	0.29
scores 0-100; high scores indicate higher health-related quality of life	30 days post-discharge	58	94.6 [83.7-100]	87 [77.2-100]	0.34
	1 year post-discharge	65	92.9 [88-98.9]	96.2 [86.4-100]	
Quality of Life- Parent Reported for Patient	At discharge	70	87 [75-93.5]	87.5 [79.3-93.5]	0.61
scores 0-100; high scores indicate higher health-related quality of life	30 days post-discharge	58	94 [83.7-100]	88.6 [78.8-98.4]	0.3
	1 year post-discharge	65	92.5 [87-98.9]	92.4 [83.2-98.4]	
Decisional conflict scale<br>scores 0-100; high scores indicate more conflict	Immediately after decision	71	0 [0-0]	0 [0-0]	0.83
Decisional regret scale	At discharge	70	40 [40-45]	40 [40-45]	0.69
scores 0100-; higher scores indicate higher regret	30 days post-discharge	66	40 [40-40]	42.5 [40-50]	0.01
Satisfaction with Decision	30 days post-discharge	59	100 [80-100]	88.3 [45-100]	0.19
	1 year post-discharge	67	100 [80-100]	100 [80-100]	0.39
Hospital Readmission	30 days post-discharge	67	0/42 (0.0)	13/25 (52.0)	<0.001
	1 year post-discharge	69	3/44 (6.8)	25/25 (100.0)	<0.001
ED/UC visit	30 days post-discharge	64	5/42 (11.9)	1/24 (4.2)	0.40
	1 year post-discharge	68	8/44 (18.2)	1/24 (4.2)	0.14

© 2019 Minneci PC et al. *JAMA Network Open*

Disability days	30 days post-discharge	66	2 [1-4]	4.5 [2-9]	0.003
	1 year post-discharge	68	2 [1-3]	13 [5.5-22]	<0.001
School days missed	30 days post-discharge	66	1 [0-2]	2 [1-3.5]	0.01
	1 year post-discharge	68	1 [0-2]	4.8 [2.5-6]	<0.001
Normal activity days missed	30 days post-discharge	66	1 [0-3]	3 [1-5]	0.01
	1 year post-discharge	68	1 [0-2]	7 [4.5-19]	<0.001
Guardian days missed from normal activities	30 days post-discharge	66	2 [1-3]	3 [2-5]	0.01
	1 year post-discharge	68	1 [1-3]	5 [3-7]	<0.001
Failure of non-operative management or recurrence of appendicitis					
Any failure/recurrence	30 days post-discharge	25	n/a	12/25 (48.0)	n/a
	1 year post-discharge	24	n/a	24/24 (100.0)	n/a
Failure/recurrence with complicated	30 days post-discharge	25	n/a	3/25 (12.0)	n/a
appendicitis	1 year post-discharge	24	n/a	3/24 (12.5)	n/a

© 2019 Minneci PC et al. *JAMA Network Open*

eReferences.

1. Hibbard JH, Stockard J, Mahoney ER, Tusler M. Development of the patient activation measure (PAM): conceptualizing and measuring activation in patients and consumers. Health Serv Res. 2004;39(4, pt 1):1005-1026. doi:10.1111/j.1475-6773.2004.00269.x
2. Ottawa Hospital Research Institute. Patient Decision Aids: Ottawa Decision Support Framework. https://decisionaid.ohri.ca/odsf.html. Modified June 22, 2015. Accessed August 15, 2012.
3. Cegala DJ, McClure L, Marinelli TM, Post DM. The effects of communication skills training on patients' participation during medical interviews. Patient Educ Couns. 2000;41(2):209-222. doi:10.1016/S0738-3991(00)00093-8
4. Cegala DJ, Post DM, McClure L. The effects of patient communication skills training on the discourse of older patients during a primary care interview. J Am Geriatr Soc. 2001;49(11):1505-1511. doi:10.1046/j.1532-5415.2001.4911244.x
5. Cegala DJ, Street RL Jr, Clinch CR. The impact of patient participation on physicians' information provision during a primary care medical interview. Health Commun. 2007;21(2):177-185. doi:10.1080/10410230701307824
6. Cegala DJ, Post DM. The impact of patients' participation on physicians' patient-centered communication. Patient Educ Couns. 2009;77(2):202-208. doi:10.1016/j.pec.2009.03.025

© 2019 Minneci PC et al. *JAMA Network Open*
