# Supplementary material for: Effects of a Patient Activation Tool on Decision Making Between Surgery and Nonoperative Management for Pediatric Appendicitis: A Randomized Clinical Trial
Source: JAMA Netw Open. 2019 Jun 7;2(6):e195009. doi: 10.1001/jamanetworkopen.2019.5009 (PMC6563561; doi:10.1001/jamanetworkopen.2019.5009)

Protocol

Randomized Controlled Trial of a Patient Activation Tool in Pediatric Appendicitis

An investigator-initiated study at Nationwide Children’s Hospital

Principal Investigator:

Katherine Deans, MD, MHSc.
Assistant Professor of Surgery
Co-Director, Center for Surgical Outcomes Research
Division of Pediatric Surgery
Nationwide Children's Hospital, Columbus, OH 43205

TABLE OF CONTENTS

Table of Contents

1. Background Information and Rationale
    - 1.1. Introduction
    - 1.2. Relevant Literature and Data

2. Study Objectives
    - 2.1. Primary Objective
    - 2.2. Secondary Objective

3. Investigational Plan
    - 3.1. Study Design
    - 3.2. Study Duration and Enrollment
        - 3.2.1. Duration of Study and Enrollment
        - 3.2.2. Total Number of Subjects Projected and Site
    - 3.3. Study Population
        - 3.3.1. Inclusion Criteria
        - 3.3.2. Exclusion Criteria

4. Study Procedures

5. Statistical Considerations
    - 5.1. Primary and Secondary Endpoints
    - 5.2. Statistical Methods
    - 5.3. Sample Size and Power

6. Study Administration
    - 6.1. Data Collection and Management
    - 6.2. Regulatory and Ethical Considerations
        - 6.2.1. Risk Assessment
        - 6.2.2. Potential Benefits of Trial Participation
        - 6.2.3. Risk-Benefit Assessment
    - 6.3 Recruitment Strategy
    - 6.4. Informed Consent/Assent
    - 6.5. Payment to Subjects/Families
    - 6.6. Confidentiality

7. References

Appendix

1. Background Information and Rationale

1.1 Introduction

Emergency surgical interventions represent stressful and difficult decisions for patients and caregivers. Approximately 136 million Americans seek emergency medical care each year with over 3.4 million people being evaluated for the diagnosis of abdominal pain. Appendectomy is the most common emergency operation performed for abdominal pain in both adults and children. Nonoperative management of appendicitis with antibiotics alone has emerged as a viable treatment alternative to appendectomy. Despite the extensive costs and morbidity of emergency surgery, as well as uncertainties about treatment, there is little evidence examining the effects of patient activation on decision making and patient centered outcomes in the emergency care setting. We propose a randomized controlled trial (RCT) of standard surgical consultation alone compared to standard surgical consultation plus a patient activation tool (PAT) in patients choosing operative or non-operative therapy for appendicitis. Our goal is to determine if a PAT can improve decision making and patient centered outcomes in pediatric patients with appendicitis and their caregivers.

1.2 Relevant Literature and Data

According to the U.S. Department of Defense Patient Safety Program, there are three essential components of patient activation: establishing patients' willingness to take an active role in their care, ensuring patients' confidence in their ability to manage their care, and providing patients with the knowledge and skills necessary to make decisions and manage their own care.[1] In adult patients, patient activation has been shown to improve healthy behaviors, increase the appropriate use of healthcare resources, improve patient preparedness for medical appointments, and improve the management and control of chronic illnesses such as diabetes.[2-8] There is little evidence examining patient activation on decision making and patient centered outcomes in the emergency care setting, or in pediatric surgery.

Emergency surgical interventions present stressful and difficult decisions for patients and caregivers. Surgeons provide generalized information about treatment options to acutely ill patients and time for shared decision making is limited. This is especially true in vulnerable populations such as children, in which caregivers are forced to take in large amounts of information and make quick decisions for someone other than themselves. We hypothesize that strategies to activate caregivers in the emergency setting through knowledge and engagement may result in improved decision making with improved self-efficacy and confidence. This could lead to improvements in other patient centered outcomes such as health related quality of life and healthcare satisfaction for both the caregiver and the pediatric patient. In this study, we define an activated patient-caregiver dyad primarily on the willingness, knowledge, engagement, and self-efficacy of the caregiver.

Our study has the potential to significantly alter patient health, well-being, and the quality of care that patients receive in four ways: First, patient activation in *pediatric* patient-caregiver dyads will be examined. The majority of studies on patient activation, decision making, and patient centered outcomes focus on autonomous adult patients.[9-13] However, the role of the caregiver is crucial in all of pediatric medicine.[14] Despite this, there are few studies that examine patient-caregiver dyads in the context of clinical decision making and even fewer that study children.[14-20] Second, this study focuses on patient activation in the emergency care setting. Most research on patient activation, decision making, and patient centered outcomes is focused on chronic illness and/or outpatient care.[9-13] Third, this study investigates the effects of patient activation on decision making and patient centered outcomes in a surgical disease. Several studies investigating the ability of decision aids to improve patient knowledge, one component of activation, have been performed in patients undergoing major elective surgery.[13] However, none of these studies targeted emergency surgical procedures or children. Results from our study will provide information on the utility of a PAT to facilitate patient-caregiver dyad activation in emergency surgical interventions and in children. Fourth, this study will use a multi-media patient activation

tool that incorporates patient activation strategies with a decision aid. While traditional paper-based tools and multi-media tools may contain identical information, the use of a multi-media tool offers the advantage of customization, feedback, and greater visual features.[21-23] In addition, multimedia has the added benefit of motivating participants to learn topics that they would usually avoid and obtain information they would not seek out from traditional sources.[24-27] Furthermore, the PAT incorporates interactive technology designed to promote patient activation, improve decision making, and effectively communicate and resolve uncertainty, in addition to eliciting rapid understanding of the disease and treatment options.

We are currently performing a pilot study offering nonoperative management as a treatment choice to patients and their caregivers. Thus far, 80% of children initially treated non-operatively did not require appendectomy, and no patients have had an adverse event or progressed in disease severity. We believe that both non-operative management and appendectomy are safe and effective treatment options for early appendicitis. As such, we are beginning to offer antibiotic therapy as an alternative treatment option to patients outside those participating in the pilot study. Although we believe we have enrolled a sufficient number of patients to determine that non-operative management is safe, we are continuing to enroll patients in the pilot study in order to more precisely determine the recurrence rate.

However, determining the best treatment requires engaging the patient and caregiver in the treatment decision. During the study, caregivers reported difficulties to our research team in understanding the treatment choices and determining which treatment choice was best for them. A previous study by our group (Kelleher) demonstrated that a computerized parent education tool improved knowledge about appendicitis in parents giving informed consent for their child to undergo appendectomy.[28] This study demonstrated the potential value of using computerized tools in the emergency surgical setting to provide information to parents about the care of their child. We hypothesize that an interactive PAT that activates and informs the child and the caregiver will lead to more willing, confident, and informed treatment choices in pediatric patients with early appendicitis and their caregivers. This activation process is expected to improve measures of decision making and patient-centered outcomes without compromising medical outcomes.

To our knowledge, this will be the first study to combine a patient activation strategy with a decision aid for patient-caregiver dyads in the emergency surgical setting. Results from this study will be informative about the utility of patient activation in this setting. A positive result from our study could lead to the rapid development of PATs for appendicitis at the majority of children's hospitals in the United States and Canada. In addition, because the underlying structure of the PAT is modular, it can be adapted and studied for use in other emergency and urgent surgical procedures in both children and adults.

2. Study Objectives

2.1 Primary Objective

Phase 1 Primary Objective: To develop an interactive PAT that will engage both parent and child in learning about appendicitis and activating the family with an expectation of improving decision making and patient-centered outcomes.

Phase 2 Primary Objective: After development of the PAT, to perform a randomized controlled trial to determine if a patient activation tool (PAT) can improve decision making and patient centered outcomes in pediatric patients with appendicitis and their caregivers, as compared to standard surgical consultation.

2.2 Secondary Objective

The secondary objective is to characterize the effects of a PAT on medical outcomes from appendicitis in patients receiving the PAT compared to those receiving standard surgical consultation alone. We will determine differences in disability days, length of stay, readmission rates, and medical complications related to treatment choice (e.g. infection, recurrence).

3. Investigational Plan

3.1 Study Design

This study will be performed in two phases.

Phase 1:
We will develop the interactive PAT in collaboration with software developers, computer scientists, and communication experts to ensure that the PAT is designed in a manner that will engage and activate patients and caregivers.

The PAT will be designed to encourage active involvement in the treatment choice, outline the two treatment options with the attendant risks and benefits, elucidate the caregiver's values and decisional support, and teach patient-caregiver dyads how to communicate decisional uncertainty.

Phase 2:
After the development of a PAT, we will conduct a prospective, single-blind, single-site, randomized trial to determine if providing patient-caregiver dyads with a PAT improves decision making and patient centered outcomes without compromising medical outcomes compared to standard surgical consultation alone in pediatric patients (7-17 years old) with early appendicitis and their caregivers. The patient-caregiver dyads and the physician research members administering the PAT will not be blinded. All clinicians subsequently interacting with these patients will be blinded as to whether the patient received the PAT or not.

3.2 Study Duration and Enrollment

Phase 2:
3.2.1 Duration of Study and Enrollment

This study will be conducted for at least 3 years with follow-up phone calls at 30 days, 6 months and 1 year, then annually for 3 years. At the conclusion of the 3 year period, for the subjects who have choosen non-operative management, an annual follow-up will be conducted.. A minimum of 3 year's data will be necessary to collect.

3.2.2. Total Number of Subjects Projected and Site

A total of 200 patient-caregiver dyads (100 in each arm) are projected to be enrolled. The study will be conducted at Nationwide Children's Hospital (NCH). Eligible patients will be recruited and consented during their initial surgical evaluation in the emergency department.

Follow-up research visits will be conducted either by phone, email, at the outpatient surgical center of Nationwide Children's Hospital (NCH), or at the Center for Surgical Outcomes Research (CSOR) within the

Center for Innovation in Pediatric Practice (CIPP) at the Research Institute at Nationwide Children's Hospital (RINCH)**.**

3.3. Study Population

Children between the ages of 7-17 who are diagnosed with early appendicitis will be screened for eligibility.

3.3.1 Inclusion Criteria

- Age : 7-17 years
- US or CT confirmed early appendicitis:
  - US: hyperemia, </=1.1 cm in diameter, compressible or non-compressible, no abscess, no fecalith, no phlegmon
  - CT: hyperemia, fat stranding, </=1.1 cm in diameter, no abscess, no fecalith, no phlegmon
- WBC < 18, >5
- Focal abdominal pain </= 48hours prior to receiving antibiotics

3.3.2 Exclusion Criteria

- Positive urine pregnancy test
- Diffuse peritonitis
- History of chronic intermittent abdominal pain

4. Study Procedures

This study will be performed in two phases.

Phase 1:
Develop the PAT in collaboration with software developers, computer scientists, and communication experts.

This will be an interactive program that will allow patients to choose an avatar who will guide them through an educational program. This will be designed for both the patient (child) and caregiver, and is meant to present information about their condition (acute appendicitis) and the treatment options (appendectomy or antibiotics). In addition to providing information regarding appendicitis and the two treatment options, the PAT will include two separate brief video clips that encourage active involvement in the treatment choice and teach patient-caregiver dyads how to communicate decisional uncertainty.

Phase 2: (Target start date 3/1/14)
Screening and Enrollment

All children age 7-17 years with a diagnosis of early appendicitis treated at NCH will be screened for study eligibility. Once eligibility is confirmed, and consent obtained, the patient-caregiver dyad is randomized to either a standard surgical consultation (control arm) or to a surgical consultation plus the PAT (intervention arm) by physician research members trained to perform both types of consultations. The consultations will occur in the NCH Emergency Department or one of the inpatient units at NCH. They will then be consented and consultation will commence.

Study Procedures:

Consultation

All randomized patients will be approached by a trained physician research member who will conduct the consultation prior to meeting the surgeon.

Patient-caregiver dyads randomized to the standard consultation will receive only verbal information regarding the two treatment options (operative or non-operative management) with patients having the opportunity to ask questions. This type of consultation is akin to a typical surgical consultation in which surgeons verbally inform patients of their options and elicit questions.

A trained physician research member will administer the PAT among patient-caregiver dyads randomized to the PAT and then answer questions about the risks and benefits of each treatment option. If the child is in 5th grade or higher, they will have the option of having their own tablet to watch the PAT.

After the physician research member has answered all patient and caregiver questions, the patient-caregiver dyad will choose either appendectomy or non-operative management. Discrepancies between the caregiver's choice and the patient's choice will be recorded, but the caregiver, by law, will make the decision. Both arms will complete computer-based questionnaires that measure patient and caregiver recall, caregiver activation, preparedness for decision making, decision self-efficacy, and decisional conflict after the treatment choice has been made. Any patient or caregiver who wishes to speak with a member of the surgical team prior to making a decision will be able to do so.

The physician research member will then inform the clinical team of the treatment choice and the surgeon will be called to speak with the family for a full discussion of the care plan and to obtain surgical consent if operative management was chosen.

The patient will be admitted to the hospital and receive care according to standard protocols for either appendectomy or non-operative management of appendicitis. Based on the results of our pilot study, we have demonstrated that non-operative management is a reasonable alternative to appendectomy for cases of early, acute appendicitis. As such, we are presenting both options as initial treatment options for early acute appendicitis to patients and their caregivers.

Before discharge, the patient and legal guardian will complete another set of questionnaires that measure: the patient's quality of life (QOL), caregiver satisfaction of care received, decision self-efficacy, decisional regret, reasons for initial choice and general socioeconomic status (SES) information. We will give English-speaking participants and families the option of conducting follow-ups via phone call and/or email. The emails will be sent from REDCap to ensure security.

A follow-up reminder letter will be mailed/ emailed/texted to all families to reiterate follow-up time points.

Follow-Up

Participants who choose non-operative management

2-5 days and 10-14 days

A member of the research team will call and/or email the family for a follow-up. Any concerns, issues or complications regarding the oral antibiotics or the study will be discussed. In addition, how many days the child missed at school and the legal guardian missed at work will also be assessed.

All participants

30 (±10) days

Depending on the child and legal guardian's preference, they will either return to NCH or a member of the research team will call or email the family for a follow-up. In addition, if they have already scheduled a follow-up appointment with their surgeon, we will attempt to schedule our appointment during the same day. The child and legal guardian will complete another set of questionnaires that measure: the patient's quality of life (QOL), caregiver satisfaction of care received, decision self-efficacy, decisional regret, satisfaction with decision and patient and caregiver recall (if patient is in the 5th grade or higher and they were given their own tablet to watch the PAT).

We will also ask questions about any problems the child has had, dependent on the treatment option, with their appendix or surgery since discharge, any other issues, and how many days the child missed school and the legal guardian missed work and any out-of-pocket expenditures (e.g. insurance copayments, and outpatient medications). This will be conducted either in person, over the phone or email. A $25 VISA® ClinCard will be provided. If the child and legal guardian prefer a phone or email follow-up, the gift card will be mailed to the legal guardian's address. A parking voucher will be provided if they decide to come back to NCH for their 30 day follow-up.

Data will also be collected from additional chart review and claims data. This will include: appendicitis recurrence with determination of simple vs. complicated appendicitis from operative and pathology reports; subsequent ED visits or physician visits; subsequent readmissions and reoperations; additional imaging, pharmaceutical, and laboratory procedures related to their chosen appendicitis treatment, if any.

6 months (±15 days)

A member of the research team will call or email the family and ask questions about any problems the child has had, dependent on the treatment option, with their appendix or surgery since their 30-day follow-up. Any recurrence or other complications will be discussed. Also, additional missed days from school and work will be assessed as well as any additional out-of-pocket expenditures (e.g. insurance copayments, and outpatient medications), if any. A $30 VISA® ClinCard will be provided after successful completion of the 6 month follow-up. The gift card will be mailed to the legal guardian's address.

Data will also be collected from additional chart review and claims data. This will include: appendicitis recurrence with determination of simple vs. complicated appendicitis from operative and pathology reports; subsequent ED visits or physician visits; subsequent readmissions and reoperations; additional imaging, pharmaceutical, and laboratory procedures related to their chosen appendicitis treatment, if any.

1 year (±30 days)

A member of the research team will call or email the family and ask questions about any problems the child has had, dependent on the treatment option, with their appendix or surgery since their 6-month follow-up. Any recurrence or other complications will be discussed. Also, additional missed days from school and work will be assessed as well as any additional out-of-pocket expenditures (e.g. insurance copayments, and outpatient medications), if any. A $35 VISA® ClinCard will be provided after successful completion of the 1 year follow-up. The gift card will be mailed to the legal guardian's address.

The child and legal guardian will also complete the PEDSQL Quality of Life Inventory: Child and Parent Report, and the legal guardian will complete the Satisfaction with Decision Scale.

Data will also be collected from additional chart review and claims data. This will include: appendicitis recurrence with determination of simple vs. complicated appendicitis from operative and pathology reports; subsequent ED visits or physician visits; subsequent readmissions and reoperations; additional imaging, pharmaceutical, and laboratory procedures related to their chosen appendicitis treatment, if any.

Subjects who turn 18 during the study, will be verbally consented.

Annually (±30 days)

A member of the research team will call or email the patient and/or family on an annual basis. This will help us determine the rates of recurrence and long term surgical complications beyond the 1 year follow up that has been performed in prior studies regarding non-operative management and appendectomy for appendicitis.

A $50 VISA® ClinCard gift card will be provided after completion of their 2 year and 3 year follow-up evaluations

Participants who choose non-operative management

For the participants who have chosen non-operative management, we will continue to contact them annually in order to access the inherent risks of choosing non-operative management.

5. Statistical Considerations

5.1. Primary and Secondary Endpoints

Patient Centered

Primary patient centered endpoints are decision self-efficacy immediately after the patient and caregiver have made their decision regarding operative or non-operative treatment (within one hour of the decision) and healthcare satisfaction at discharge.

Secondary patient centered endpoints are preparedness for decision making and caregiver activation level immediately after the patient and caregiver have made their decision, recall (knowledge) immediately after the patient and caregiver have made their decision and at 30 days follow-up, decision regret at discharge and 30 days follow-up, decisional conflict immediately after the patient and caregiver have made their decision, reasons for initial choice at discharge, satisfaction with decision and health care associated costs at 30 days and 1 year, and health related quality of life (HRQOL) prior to discharge and 30 days after discharge.

Medical

The primary medical endpoint is disability days at 1 year. Disability days is a composite of inpatient hospital days, emergency department visits, primary care physician visits and all days with limited activities referable to their appendicitis.

Secondary endpoints are disability days at 30 days and 6 months, readmission rate and length of stay. We also hope to determine the rate of recurrence of acute appendicitis for those patients who opt to receive antibiotic therapy and the rate of surgical complications for patients undergoing appendectomy. This outcome will be measured at the aforementioned time points, and will also be measured on an annual basis.

5.2. Statistical Methods

Patient-centered endpoints to be analyzed using mixed models across all time points at which the measure was determined are total decision self-efficacy scores, total PedsQL™ healthcare satisfaction generic module scores, and total PedsQL™ HRQOL general core scale scores in patients and caregivers. Time will be included as a categorical variable in each of these models, and its interaction with the PAT intervention will also be included. The interaction terms will provide estimates of the differences in these patient-centered outcomes between PAT and standard surgical consultation arms at each time point in the study. An unstructured error covariance approach will be used to account for the within-subject correlation in outcomes measured over time.

For disability days in a year, we will test for non-inferiority with a margin of less than two days. It is possible that the PAT will reduce disability days. The non-inferiority test can be followed by a superiority test for disability days, with no effect on Type I error. The PAT and standard surgical consultation groups will also be compared on their average readmission rates. We will assume a negative binomial distribution using GENMOD procedure in SAS 9.3 to model the count of days.

Subgroup Analyses

We will perform exploratory subgroup analyses to examine outcomes that are relevant only to either the operative or non-operative treatment groups. This will be especially useful if we find a difference in disability days between the randomized groups, because the difference may be related to differences in the percentage of patients within the PAT group compared to the standard surgical consultation group that choose operative vs. non-operative management.

Within the subgroup of patients that choose operative therapy, we will compare post-operative infections, re-operation rate at one year, and readmission rate at one year to identify if receiving the PAT altered these medical outcomes.

Within the subgroup of patients who chose non-operative therapy, we will examine the need for appendectomy during initial admission, recurrence of appendicitis at one year, and antibiotic side effects at 30 days to identify if receiving the PAT altered these outcomes. Proportions will be compared for antibiotic complications, recurrence, post-operative infection, and complications. Average readmission rates will be compared using a Pearson chi-square test.

5.3. Sample Size and Power

Our sample size is 200 total patient-caregiver dyads.

The primary patient centered outcomes are decision self-efficacy immediately after the patient and caregiver have made their choice regarding operative or non-operative treatment (within one hour of the intervention) and healthcare satisfaction at discharge. We expect the PAT will increase the total Decision Self- Efficacy Scale score by 0.4 standard deviations, and increase total PedsQL™ healthcare satisfaction generic module scores at discharge by 0.5 standard deviations.[29-30] Assuming that we will show superiority for one or both of these patient centered outcomes, we also consider it critical to claim that the PAT does not increase the number of disability days over a year as compared to standard surgical consultation. Based on our previous research and published data, we expect the number of disability days in the standard surgical consultation group to be 14 days with a standard deviation of 5 days.[31] Based on this, we set our noninferiority margin to 14%, or two days more. For the primary patient centered outcomes we will use Holm's procedure to test the two primary outcomes. Non-inferiority of disability days will only be meaningful if we achieve significance on these

primary outcomes. Given this, we plan to use a gatekeeping fallback procedure, whereby we can test for non-inferiority at the one-sided 0.025 level when we achieve significance for one or both of the patient centered primary outcomes.[32] If we fail to reach significance on these primary outcomes, then we will have to use a much more stringent alpha of 0.005 (fallback). In order to use this procedure, our significance level for the patient centered primary outcomes will be set at 0.02 (rather than 0.025).

Based on this strategy, 100 patients in each group will provide power of 92% to reach significance on either primary patient centered outcome, and 74% power to reach significance on both, assuming the effect sizes listed above. This sample number provides 80% power to claim significant non-inferiority of less than two days. Over 200 patients who meet eligibility criteria for the proposed study are admitted to Nationwide Children's Hospital (NCH) annually. Therefore, a 50% annual recruitment rate should ensure adequate enrollment.

6. Study Administration

6.1. Data Collection and Management

All clinical monitoring performed as standard procedure in the hospital, such as vital signs, pain scoring, and dietary advancement, and other pertinent variables for the purposes of the study, will be recorded. Standardized surveys and internally created follow-up surveys will also be recorded. Study staff will collect, compile and manage this data. This data will be compiled on REDCAP to which only study staff will have access.

6.2. Regulatory and Ethical Considerations

6.2.1. Risk Assessment

Loss of confidentiality could be a risk.

Randomization

The randomized arms of this study include a standard surgical consultation or a consultation with a PAT.

Standard Consultation

There are no increased risks to the standard surgical consultation group. However, the surveys used to assess decision making and patient centered outcomes may make the subjects feel uncomfortable due to the inclusion of questions that ask about their emotional and social functioning.

Consultation with PAT

Potential risks may include increases in decisional conflict or regret based on being activated and receiving information that might lead to more stress about their decision or may lead them to a decision they may not have made prior to viewing the PAT. In addition, the surveys used to assess decision making and patient centered outcomes may make the subjects feel uncomfortable due to the inclusion of questions that ask about their emotional and social functioning.

Hospital Course

The inherent risks of choosing non-operative management or appendectomy. For non-operative management, patients may require surgery and for appendectomy, patients may develop intra- and post-operative complications.

In addition, the surveys used to assess decision making and patient centered outcomes may make the subjects feel uncomfortable due to the inclusion of questions that ask about their emotional and social functioning.

6.2.2. Potential Benefits of Trial Participation

Randomization

Patients randomized to receive the PAT may directly benefit from receiving this intervention with potential improvements in: (1) measures of decision making including improved knowledge and self-efficacy and decreased conflict and regret; and, (2) patient centered outcomes, such as satisfaction and health related quality of life.

There are no direct benefits to patients randomized to standard surgical consultation but information obtained may help others.

Potential benefits of this research include determining that patient-caregiver activation for pediatric appendicitis improves decision making and patient centered outcomes. The results of this study can be used to inform both pediatric and adult clinical practice on the value of incorporating patient activation in emergency surgical care and would potentially benefit many future patients with appendicitis and other emergency surgical diseases.

Hospital Course

Non-Operative Group
The potential benefits of non-operative management include avoiding surgery, less pain, and quicker return to normal activities.

Surgery Group
The potential benefit of an appendectomy is that it is curative for appendicitis.

6.2.3. Risk-Benefit Assessment/Risk Minimization

All patients will be informed that their participation in this study is voluntary and that their medical care will be unchanged whether they agree or do not agree to participate in this study. Further, they will be informed that if they agree to enter this study, they may withdraw from the study at any time.

Privacy of individuals and confidentiality of data will be maintained by minimizing the amount of identifiable data obtained as much as possible. Only study IDs will be used to identify patients on all data forms and in all datasets used for analysis. The file linking study IDs to patient names and MRN will be password protected and will not be made available to non-study staff or used during data analysis. All information will be compiled and stored in REDCAP and will be password protected. Strict standards of confidentiality will be upheld at all times.

All patients enrolled in the trial will be monitored according to the standard care protocol for their

chosen treatment option (operative or non-operative management). Any adverse events will be handled according to hospital procedure. Before discharge, patients and their caregivers will be given clear instructions on taking either oral antibiotics or pain medicines and for reporting any concerns while on these medications. A research phone number will be provided in the informed consent should they have any questions or concerns relating to the study and contact information for medical questions will be provided consistent with the standard of care for surgical discharges at our institution. After hospital discharge, any further complications will also be assessed. Participants may call the research staff at any time to address concerns and a physician member of the research team is available to provide medical advice. This will be stated in the informed consent.

Regarding the patient centered surveys, participants have the option of skipping questions they find to be uncomfortable or simply wish to refuse answering. This will be stated on the informed consent

6.2.4. Data safety and monitoring

This study is only minimal risk. Data will be monitored by research team members once a week. They will ensure that all data (clinical data and questionnaires) collected are correctly completed. The clinical study team will review adverse events as they occur.

6.2.5. Adverse events (AE) and Serious adverse events (SAEs)

Adverse events which may occur during this study include increases in decisional conflict or regret based on being activated and receiving information that might lead to more stress about their decision or may lead them to a decision they may not have made prior to viewing the PAT. In addition, the surveys used to assess decision making and patient centered outcomes may make the subjects feel uncomfortable due to the inclusion of questions that ask about their emotional and social functioning. Several adverse events may occur as part of the treatment choice that are not related to enrollment in the trial: All patients could have adverse reactions to the antibiotics; patients who choose non-operative management could have interval progression of appendicitis or the need to be readmitted due to recurrence of disease; and patients that choose operative management could have intra-operative or post-operative complications such as bleeding and abscesses. These are all expected complications that will not be reported to the IRB.

We do not expect any severe AE in either study arm. All unexpected non-serious adverse events and serious adverse events relating to the administration of the PAT will be reported verbally and in writing to PCORI and the NCH IRB chair. The verbal report will occur within 48 hours of the occurrence. The written report of the serious adverse event (e.g., death or life-threatening adverse event) will be reported within seven days.

6.3. Recruitment Strategy

No active recruitment will take place. Subjects will be screened for eligibility once a diagnosis of early appendicitis has been determined and the surgical consult team has been contacted.

Eligible patients are identified by the emergency department (ED), who is typically responsible for the initial diagnostic evaluation of patients presenting with abdominal pain. The ED staff will be educated about this study and will be empowered to directly contact the study staff about eligible patients. Similarly, the surgical

staff (residents, nurse practitioners, and attending surgeons) will also be fully informed about the study and will be able to notify study staff of a new eligible patient, either in the ED or arriving at the hospital as a direct admission from an outside institution.

6.4. Informed Consent/Assent

All informed consents and assents will be performed by a clinically trained member (physician) of the research team.

Refusal to participate in the study or withdrawal from the study will not affect the child's receipt of clinical care.

6.5. Payment to Subjects/Families

The parent/legal guardian of all participants will be paid a $25 VISA® ClinCard on their 30 day, $30 on their 6 month and $35 on their 1 year follow-up visit. If the child and legal guardian choose phone or email follow-up, the gift card will be mailed to the legal guardian's address. A parking voucher will be provided if the family chooses to come back to NCH for their 30 day follow-up. A $50 VISA® ClinCard will be provided after completion of their 2 year and 3 year follow-up evaluations.

6.6. Confidentiality

Privacy and security will be maintained by minimizing the amount of identifiable data as much as possible. Only study identifications (IDs) will be used to identify patients on all data forms and all datasets used for analysis. The file linking study IDs to patient names and medical record numbers (MRNs) will be password protected and will not be made available to non-study staff or used during data analysis. All information will be compiled in REDCAP, to which only study staff will have access.

7. References

1. US Department of Defense. Module 1: The Patient Activation Continuum. Patient Activation Reference Guide: Web Page. Available at: http://www.health.mil/dodpatientsafety/productsandservices/parg.aspx. March 2012. Accessed October 26, 2012.
2. Greene J, Hibbard JH, Tusler M. How much do health literacy and patient activation contribute to older adults' ability to manage their health?: AARP Public Policy Institute; 2005.
3. Hibbard JH, Cunningham PJ. How engaged are consumers in their health and health care, and why does it matter. Res Briefs. 2008;8:1-9.
4. Hibbard JH, Greene J, Becker ER, et al. Racial/ethnic disparities and consumer activation in health. Health Aff (Millwood). Sep-Oct 2008;27(5):1442-1453.
5. Mosen DM, Schmittdiel J, Hibbard J, Sobel D, Remmers C, Bellows J. Is patient activation associated with outcomes of care for adults with chronic conditions? J Ambul Care Manage. Jan-Mar 2007;30(1):21-29.
6. Rask KJ, Ziemer DC, Kohler SA, Hawley JN, Arinde FJ, Barnes CS. Patient activation is associated with healthy behaviors and ease in managing diabetes in an indigent population. The Diabetes educator. Jul-Aug 2009;35(4):622-630.
7. Remmers C, Hibbard J, Mosen DM, Wagenfield M, Hoye RE, Jones C. Is patient activation associated with future health outcomes and healthcare utilization among patients with diabetes? J Ambul Care Manage. Oct-Dec 2009;32(4):320-327.
8. Skolasky RL, Green AF, Scharfstein D, Boult C, Reider L, Wegener ST. Psychometric properties of the patient activation measure among multimorbid older adults. Health Serv Res. Apr 2011;46(2):457-478.
9. Anderson LA, Sharpe PA. Improving patient and provider communication: A synthesis and review of communication interventions. Patient Education and Counseling. 1991;17(2):99-134.
10. Harrington J, Noble LM, Newman SP. Improving patients' communication with doctors: a systematic review of intervention studies. Patient Educ Couns. Jan 2004;52(1):7-16.
11.Haywood K, Marshall S, Fitzpatrick R. Patient participation in the consultation process: a structured review of intervention strategies. Patient Educ Couns. Oct 2006;63(1-2):12-23.
12.. Post DM, Cegala DJ, Miser WF. The other half of the whole: teaching patients to communicate with physicians. Fam Med. May 2002;34(5):344-352.
13.. Stacey D, Bennett CL, Barry MJ, et al. Decision aids for people facing health treatment or screening decisions. Cochrane Database Syst Rev. 2011(10):CD001431.
14.. Pennarola BW, Rodday AM, Mayer DK, et al. Factors associated with parental activation in pediatric hematopoietic stem cell transplant. Med Care Res Rev. Apr 2012;69(2):194-214.
15. Harrington NG, Norling GR, Witte FM, Taylor J, Andrews JE. The effects of communication skills training on pediatricians' and parents' communication during "sick child" visits. Health Commun. 2007;21(2):105-114.
16. Lewis CC, Pantell RH, Sharp L. Increasing patient knowledge, satisfaction, and involvement: randomized trial of a communication intervention. Pediatrics. Aug 1991;88(2):351-358.
17. Coyne I, Gallagher P. Participation in communication and decision-making: children and young people's experiences in a hospital setting. J Clin Nurs. Aug 2011;20(15-16):2334-2343.
18. Jackson C, Cheater FM, Reid I. A systematic review of decision support needs of parents making child health decisions. Health Expect. Sep 2008;11(3):232-251.
19. Lipstein EA, Brinkman WB, Britto MT. What is known about parents' treatment decisions? A narrative review of pediatric decision making. Med Decis Making. Mar-Apr 2012;32(2):246-258.
20. Moore L, Kirk S. A literature review of children's and young people's participation in decisions relating to health care. J Clin Nurs. Aug 2010;19(15-16):2215-2225.
21. Lakhmani S, Bowers C. Reflected in a liquid crystal display: personalization and the use of avatars in serious games. Virtual and Mixed Reality-Systems and Applications. 2011:237-242.
22.Lieberman DA. Management of chronic pediatric diseases with interactive health games: theory and research findings. J Ambul Care Manage. Jan 2001;24(1):26-38.
23.Sheehan J, Sherman KA. Computerised decision aids: a systematic review of their effectiveness in facilitating high-quality decision-making in various health-related contexts. Patient Educ Couns. Jul 2012;88(1):69-86.

24. Austin E. Reaching Young Audiences: Developmental Considerations in Designing Health Messages. In: Maibach EW, Parrott RL, eds. Designing health messages: Approaches from communication theory and public health practice. Thousand Oaks, CA: Sage Publications; 1995:114-144.
25. Deardorff WW. Computerized health education: a comparison with traditional formats. Health education quarterly. Spring 1986;13(1):61-72.
26. Gustafson DH, Bosworth K, Chewning B, Hawkins RP. Computer-based health promotion: combining technological advances with problem-solving techniques to effect successful health behavior changes. Annu Rev Public Health. 1987;8:387-415.
27. Lepper MR, Gurtner JL. Children and computers. Approaching the twenty-first century. The American psychologist. Feb 1989;44(2):170-178.
28..Nwomeh BC, Hayes J, Caniano DA, Upperman JS, Kelleher KJ. A parental educational intervention to facilitate informed consent for emergency operations in children. The Journal of surgical research. Apr 2009;152(2):258-263.
29. Fraenkel L, Rabidou N, Wittink D, Fried T. Improving informed decision-making for patients with knee pain. J Rheumatol. Sep 2007;34(9):1894-1898.
30. Zlowodzki M, Bhandari M. Outcome measures and implications for sample-size calculations. J Bone Joint Surg Am. May 2009;91 Suppl 3:35-40.
31. Mason RJ, Moazzez A, Sohn H, Katkhouda N. Meta-analysis of randomized trials comparing antibiotic therapy with appendectomy for acute uncomplicated (no abscess or phlegmon) appendicitis. Surgical infections. Apr 2012;13(2):74-84.
32. Wiens BL, Dmitrienko A. The fallback procedure for evaluating a single family of hypotheses. J Biopharm Stat. 2005;15(6):929-942.

Figure 1. Study Design

V:6/23/14

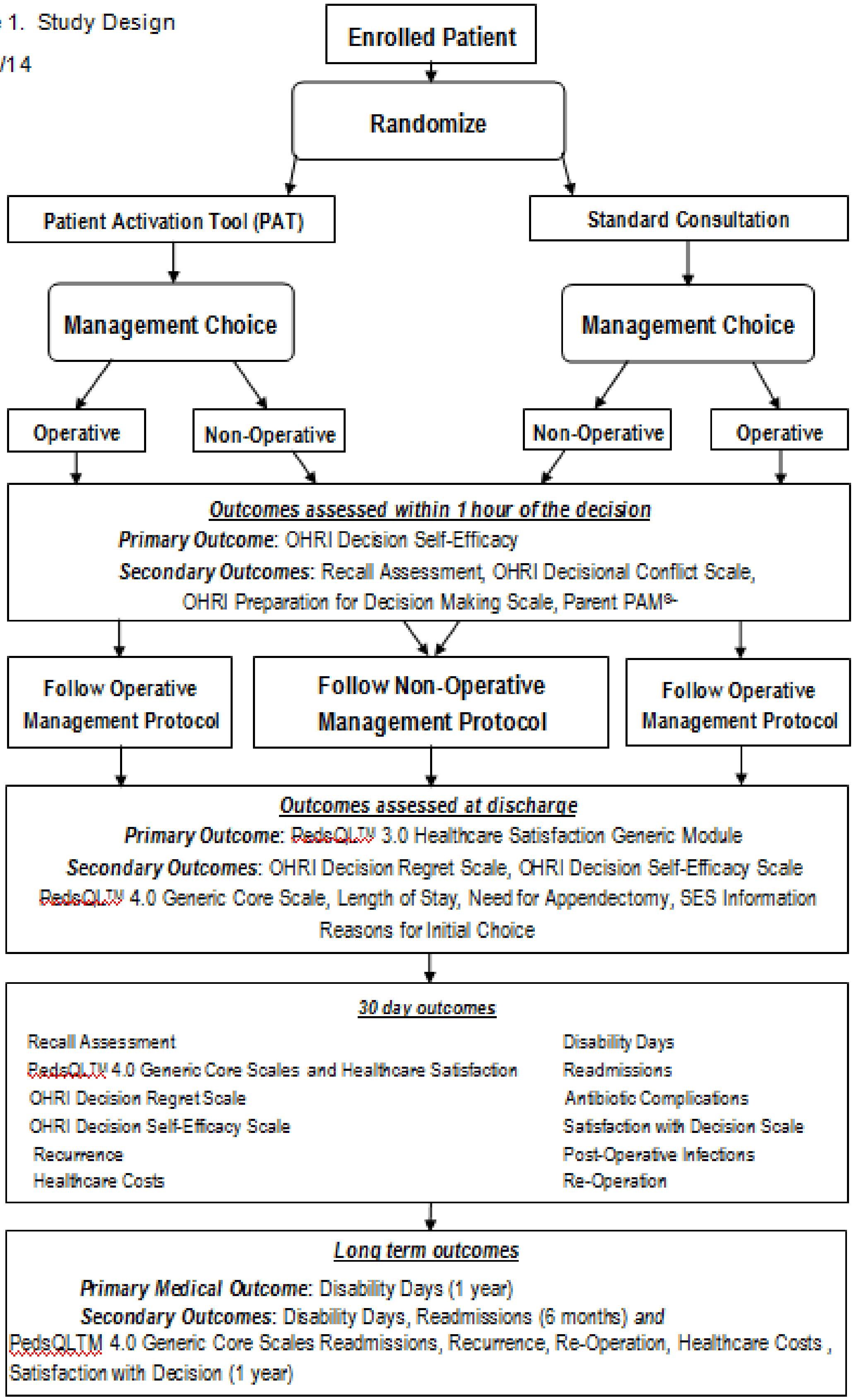

Supplement: Supplement 2. — Trial Protocol [file jamanetwopen-2-e195009-s002.pdf]
