# Supplementary material for: Effects of a Patient Activation Tool on Decision Making Between Surgery and Nonoperative Management for Pediatric Appendicitis: A Randomized Clinical Trial
Source: JAMA Netw Open. 2019 Jun 7;2(6):e195009. doi: 10.1001/jamanetworkopen.2019.5009 (PMC6563561; doi:10.1001/jamanetworkopen.2019.5009)
Supplement: Supplement 3. — Data Sharing Statement [file jamanetwopen-2-e195009-s003.pdf]

Data Sharing Statement

Minneci. Effects of a Patient Activation Tool on Decision Making Between Surgery and Nonoperative Management for Pediatric Appendicitis. *JAMA Netw Open*. Published June 07, 2019. 10.1001/jamanetworkopen.2019.5009

Data
Data available: Yes
Data types: Deidentified participant data, Data dictionary
How to access data: Katherine.deans@nationwidechildrens.org
When available: With publication

Supporting Documents
Document types: None

Additional Information
Who can access the data: Researchers whose proposed use of the data has been approved
Types of analyses: For a specified purpose
Mechanisms of data availability: after approval of a proposal, or with a signed data access agreement
